# Anticoagulation use in rheumatic heart disease-associated atrial fibrillation: a study of real-world practice in Central Australia^☆^

**DOI:** 10.1016/j.ijcha.2025.101711

**Published:** 2025-05-30

**Authors:** Sonia Sawant, Gabrielle Freedman, Ana Garcia, Sally Terrett, Chinmay Khandkar, Anosh Sivashanmugarajah, Angus Baumann

**Affiliations:** a Department of Cardiology, Alice Springs Hospital, Alice Springs, Australia; b Department of Cardiology, St Vincent’s Hospital Sydney, Sydney, Australia; c Sydney Medical School, the University of Sydney, Sydney, Australia; d School of Public Health, Imperial College London, London, United Kingdom; e Department of Cardiology, Royal Prince Alfred Hospital, Sydney, Australia; f Flinders University Rural Clinical School, Flinders University, Adelaide, Australia

**Keywords:** Rheumatic heart disease, Atrial fibrillation, Anticoagulation, Stroke

## Abstract

**Background:**

Rheumatic heart disease (RHD) disproportionately impacts minority groups. Indigenous Australians living in remote areas such as Central Australia account for 3.8% of the population and 92% of RHD cases. A complication of RHD is valvular atrial fibrillation (vAF). Previous studies favour Vitamin K antagonist (VKA) use in vAF over direct oral anti-coagulants (DOACs). However, challenges to VKA use remain. This real-world retrospective observational study aimed to compare clinical outcomes between patients prescribed VKAs or DOACs for vAF in Central Australia over a five-year period.

**Methods:**

Patients with RHD and vAF on the Northern Territory RHD Register in January 2019 were identified and five-year outcome data collected. Patients were grouped and analysed according to prescribed oral anticoagulant therapy in January 2019 (intention to treat (ITT)) or in January 2024/time of event (as-treated analysis (AT)). The primary outcome was major adverse cardiac and cerebrovascular events (MACCE). The safety endpoint was major bleeding.

**Results:**

Of patients with vAF, 49 were included in the ITT analysis and 51 in the AT analysis. The mean age was 61.9 ± 13.9 years and 68.9 % were female. There was no difference in MACCE (25.0 % vs 22.2 %, p = 0.86) or major bleeding (20.0 % vs 11.1 %, p = 0.53) between VKAs and DOACs in the ITT analysis. Findings were similarly non-significant in AT analysis.

**Conclusion:**

This study demonstrates no significant advantage to VKA over DOAC therapy in vAF in a small cohort of RHD patients living in remote Australia. Further investigation is required to optimise treatment strategies in this important group.

## 1 Introduction

Rheumatic heart disease (RHD) predominantly impacts low/middle-income countries, with Oceania, South Asia and sub-Saharan Africa having the highest rates of RHD-associated mortality [1,2]. In Australia in 2021 nearly 10,000 people had acute rheumatic fever or RHD with Indigenous Australians accounting for 92 % of cases, many residing in remote areas [3], highlighting that minority groups in high-income countries are also at risk.

Atrial fibrillation (AF) predisposes patients to cardio-embolic events. Valvular AF (vAF) is defined as AF with mechanical heart valves or at least mild mitral stenosis, though the exact parameters vary [[4], [5], [6]]. RHD is the leading cause of mitral stenosis with a prevalence in RHD of roughly 33 % based on a *meta*-analysis of 83 studies from 42 countries [2,7].

Meta-analyses of randomised clinical trials have demonstrated similar efficacy between direct oral anticoagulants (DOACs) and Vitamin K antagonists (VKAs) for stroke prevention in non-valvular AF [[8], [9], [10]]. There have been few studies investigating DOACs in vAF. The randomised noninferiority INVICTUS trial evaluating the efficacy of Rivaroxaban compared with VKA in RHD–associated vAF across Africa, Asia and Latin America demonstrated a lower composite of stroke, systemic embolism, myocardial infarction and death with VKA [4]. Key limitations included open-label design, protocol changes and frequent physician interactions in the VKA group. Nonetheless, current guidelines recommend VKAs for the treatment of vAF [5,6].

The remote region of Central Australia has a high prevalence of RHD and vAF. Challenges to prescribing VKAs include a linguistically diverse patient population complicating effective communication, vast distances between communities and healthcare, and difficulty staffing remote clinics, impacting adherence [11,12]. Therefore, patients are often prescribed Rivaroxaban for vAF to avoid harms associated with sub-therapeutic use of VKAs which include stroke, systemic embolism and mortality [13]. Given the attractiveness of DOACs in remote settings and available evidence, we aimed to conduct a real-world retrospective observational study to examine the efficacy of DOACs compared to VKAs in patients with RHD-associated vAF in Central Australia.

## 2 Methods

### 2.1 Trial design

This retrospective observational study included patients with vAF, defined as those patients with a mechanical heart valve or at least mild mitral stenosis, on 1st January 2019 on the Northern Territory RHD register. Our definition is based on that used in the INVICTUS trial and as a result is more inclusive of milder mitral stenosis cases than major clinical guidelines [5,6]. Baseline characteristics, anticoagulation, adherence, comorbidities, echocardiographic data and five-year outcome data were collected. The study was approved by the Human Research Ethics Committee of the Northern Territory Department of Health and Menzies School of Health Research (HREC no. 2023–4780).

### 2.2 Primary and secondary outcomes

The primary outcome was major adverse cardiac and cerebrovascular events (MACCE), a composite of ischaemic stroke, systemic embolism, non-fatal myocardial infarction, cardiovascular death and all-cause mortality at five years. Secondary outcomes included all-cause mortality and ischaemic stroke. The primary safety endpoint was major bleeding.

### 2.3 Statistical analyses

**Fig. 1:**
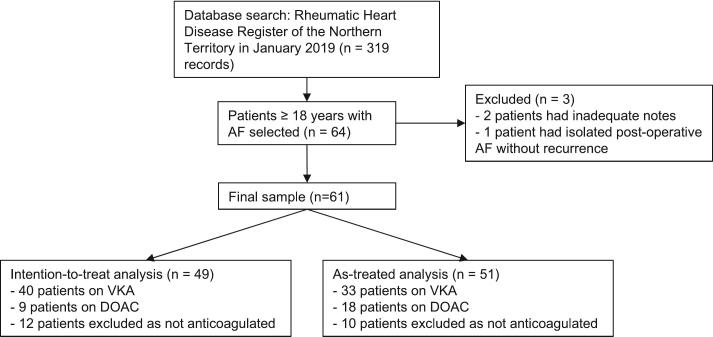
Flowchart outlining participant selection. Distribution of patients on vitamin K antagonists (VKA) and direct oral anticoagulants (DOAC) are also displayed in both intention-to-treat and as-treated analyses.

Due to cross over, statistical analyses were conducted as both intention-to-treat (ITT) based on initial anticoagulation and as-treated (AT), defined as treatment at the time of first event or at the end of follow-up if no event occurred. Patients not on anticoagulation were excluded (Fig. 1).

Nominal variables were presented as number and percentage. Normally distributed linear variables were presented as mean +/- standard deviation. Non-normally distributed variables were presented as median +/- interquartile range. Categorical variables were compared using a Pearson Chi Squared analysis while continuous, normally distributed variables were analysed by student’s *t*-test or one-way ANOVA analysis. Continuous non-normally distributed variables were analysed by Mann-Whitney or Kruskal Wallis tests. Data were analysed using SPSS v25.

## 3 Results

### 3.1 Study and patient characteristics

61 patients ≥ 18 years were included in the study (Fig. 1). At baseline, 65.6 % received VKA, 14.8 % DOAC and 19.7 % were not anticoagulated. The mean age was 61.9 ± 13.9 years and 68.9 % were female. ITT analysis included 49 patients, 40 receiving VKA and 9 receiving DOAC. AT analysis included 51 patients, 33 receiving VKA and 18 receiving DOAC. Baseline characteristics between groups were similar, though patients receiving VKA were more likely to have had previous mitral valve (MV) interventions (62.5 % vs 22.2 %, p = 0.028 in ITT; 69.7 % vs 27.8 %, p = 0.004 in AT). In the AT analysis, patients treated with VKA were younger and had lower CHADS2-Vasc scores (Table 1). Excluding those with previous mechanical MV replacement, the severity of mitral stenosis was mild (median area 1.6 cm^2^ vs 1.7 cm^2^, p = 0.80 in ITT; 1.6 cm^2^ vs 1.7 cm^2^, p = 0.66 in AT) (Table 1). 30.6 % patients crossed over from the ITT analysis, with a non-significant trend favouring switch to DOAC (11 vs 0, p = 0.09)

**Table 1:** **Baseline characteristics of patients and clinical outcomes.** Baseline characteristics and outcomes are displayed by intention to treat and as-treated analyses. Mitral stenosis (MS) severity was categorised as nil (0), mild (1), moderate (2) and severe (3), excluding those with mitral valve replacement (MVR). The primary efficacy outcome was major adverse cardiac and cerebrovascular events (MACCE) which was a composite of ischaemic stroke, systemic embolism, non-fatal myocardial infarction and all-cause mortality. Secondary efficacy outcomes included all-cause mortality and ischaemic stroke. The primary safety outcome was major bleeding. The number of patients crossing over between vitamin K antagonist (VKA) and direct oral anticoagulant (DOAC) groups are also shown.

Baseline characteristics			
Intention to treat analysis (n = 49)	VKA (40)	DOAC (9)	p-value
Age – mean (+/- SD)	60.9 (+/- 14.7)	66.8 (+/- 15.7)	0.29
Male – no. (%)	10 (25.0)	3 (33.3)	0.61
In Alice Springs – no. (%)	13 (32.5)	4 (44.4)	0.50
In Alice Springs and Tennant Creek – no. (%)	15 (37.5)	4 (44.4)	0.70
Heart failure with reduced ejection fraction – no. (%)	12 (30.0)	3 (33.3)	0.84
Prior cerebrovascular event – no. (%)	8 (20.0)	4 (44.4)	0.12
Previous major bleed – no. (%)	2 (5.0)	1 (11.1)	0.49
Chronic kidney disease stage IV – no. (%)	4 (10.0)	1 (11.1)	0.92
Aspirin use – no. (%)	3 (7.5)	1 (11.1)	0.72
Previous mitral valve intervention – no. (%)	25 (62.5)	2 (22.2)	**0.028**
Mechanical MVR – no. (%)	15 (37.5)	0 (0.0)	0.10
MS severity (cm^2^) (MVR excluded) – Median, IQR (n = 32)	1.6 (1.2, 2.1)	1.7 (1.2, 2.0)	0.80
Mean CHADS2 Vasc	3.6 (+/- 1.9)	4.8 (+/- 2.4)	0.10

Outcomes			

Intention to treat analysis (n = 49)	VKA (40)	DOAC (9)	p-value

MACCE – no. (%)	10 (25.0)	2 (22.2)	0.86
All-cause mortality – no. (%)	11 (27.5)	3 (33.3)	0.73
Ischaemic stroke – no. (%)	4 (10.0)	0 (0.0)	0.32
Major bleeding – no. (%)	8 (20.0)	1 (11.1)	0.53
Change of agent	14 (35.0)	1 (11.1)	0.16

Baseline characteristics			

As-treated analysis (n = 51)	VKA (33)	DOAC (18)	p-value

Age – mean (+/- SD)	58.6 (+/- 12.7)	68.7 (+/- 16.0)	**0.017**
Male – no. (%)	10 (30.3)	4 (22.2)	0.54
In Alice Springs – no. (%)	10 (30.3)	7 (38.9)	0.53
In Alice Springs and Tennant Creek – no. (%)	12 (36.4)	7 (38.9)	0.86
Heart failure with reduced ejection fraction – no. (%)	8 (24.2)	7 (38.9)	0.27
Prior cerebrovascular event – no. (%)	6 (18.1)	6 (33.3)	0.22
Previous major bleed – no. (%)	1 (3.0)	2 (11.1)	0.24
Chronic kidney disease stage IV – no. (%)	2 (6.1)	2 (11.1)	0.52
Aspirin use – no. (%)	2 (6.1)	1 (5.6)	0.94
Previous mitral valve intervention – no. (%)	23 (69.7)	5 (27.8)	**0.004**
Mechanical MVR – no. (%)	15 (45.5)	0 (0.0)	**0.008**
MS severity (cm^2^) (MVR excluded) – median, IQR (n = 34)	1.6 (1.2, 2.0)	1.7 (1.3, 2.2)	0.66
Mean CHADS2 Vasc	3.2 (+/- 1.8)	4.7 (+/- 2.1)	**0.013**

Outcomes			

As-treated analysis (n = 51)	VKA (33)	DOAC (18)	p-value

MACCE – no. (%)	9 (27.3)	3 (16.7)	0.39
All-cause mortality – no. (%)	7 (21.2)	6 (33.3)	0.34
Ischaemic stroke – no. (%)	5 (15.2)	0 (0.0)	0.08
Major bleeding – no. (%)	6 (18.2)	4 (22.2)	0.73

Abbreviations used: VKA = Vitamin K antagonist, DOAC = Direct oral anti-coagulant, MVR = mitral valve replacement, MS = Mitral Stenosis, MACCE = major adverse cardiac and cerebrovascular events.

### 3.2 Efficacy outcomes

ITT analysis revealed no difference in MACCE between VKAs and DOACs (25.0 % vs 22.2 %, p = 0.86) (Table 1). Similarly, AT analysis showed no difference in MACCE between VKAs and DOACs (27.3 % vs 16.7 %, p = 0.39) (Table 1).

ITT analysis showed no difference in all-cause mortality (27.5 % vs 33.3 %, p = 0.73) and ischaemic stroke (10.0 % vs 0.0 %, p = 0.32) between VKAs and DOACs (Table 1). AT analysis also showed no difference in all-cause mortality (21.2 % vs 33.3 %, p = 0.34) and ischaemic stroke (15.2 % vs 0.0 %, p = 0.08) between VKAs and DOACs (Table 1). In the AT analysis, three patients who began on no anticoagulation were on VKA at the time of MACCE.

### 3.3 Primary safety outcome

There was no difference in major bleeding between VKAs and DOACs based on ITT (20.0 % vs 11.1 %, p = 0.53) and AT analyses (18.2 % vs 22.2 %, p = 0.73) (Table 1).

## 4 Discussion

RHD disproportionately impacts minority groups globally. Lack of population-specific data have led to guidelines supporting VKAs for RHD-associated vAF which presents a challenge as individuals in remote environments are unable to reliably access healthcare [11,12]. Patients are often prescribed DOACs to allow stable dosing and avoid harms associated with sub-therapeutic VKA dosing [13]. The INVICTUS trial demonstrated a lower composite of stroke, systemic embolism, myocardial infarction and death with VKA compared to Rivaroxaban. However, the results should be interpreted with caution [4]. A key potential issue included amendment to the primary endpoint from ischaemic stroke to a composite endpoint. This was due to low event rates in each arm, possibly due to efficacy of both anticoagulants, a low risk cohort or both. The superiority of VKA was driven by lower all-cause mortality (primarily fewer sudden cardiac death and pump failure events). Due to the open-label design, patients on VKAs had frequent physician interactions for INR monitoring which may have contributed to this mortality benefit. Statistical significance for stroke reduction with VKAs was only reached in the AT analysis. The ITT analysis showed significant reduction in ischaemic stroke, but not all strokes, with VKA therapy. Finally, such adherence to VKA therapy (96.4 % over a four year period) would be difficult to achieve in resource-poor contexts associated with RHD, including Central Australia. As a result, we believe clinical equipoise still exists around the role of DOACs as an effective real-world alternative to VKAs for stroke prevention in vAF.

In our cohort, we demonstrated female predominance with a mean age of 61.9 years, consistent with previous data [7,14,15]. AF prevalence amongst those with RHD was approximately 20 %, lower than the worldwide prevalence of 33 %(2, 7). 21.3 % patients had a prior cerebrovascular event with unknown anticoagulation status during the event. Our definition of vAF was similar to INVICTUS, where vAF was defined as RHD and mitral stenosis with either CHA_2_DS_2_-VASc 2 or valve area <2.0 cm^2^. This was milder than ARISTOTLE and ROCKET-AF which excluded patients with vAF, defined as moderate-to-severe or haemodynamically significant mitral stenosis [16,17].

The comparative incidence of MACCE, all-cause mortality and ischaemic stroke between groups suggests that DOACs may have similar efficacy to VKAs. The lack of difference in major bleeding is also supportive. Numerous participants crossed over from VKAs to DOACs during the five-year period, probably reflecting a real-world change in clinical practice despite existing guidelines however we were unable to obtain information on why patients crossed over and there may be confounding factors that introduce a potential bias.

In recent years, studies have explored the use of DOACs in vAF. A retrospective cohort study of 56,336 patients with vAF showed that DOACs were associated with lower risk of ischaemic stroke or systemic embolism and major bleeding in comparison with VKA [18]. The RISE-MS trial studied DOACs for vAF with moderate-to-severe mitral stenosis and demonstrated no difference in thromboembolic and bleeding events compared to VKA [19]. A *meta*-analysis of clinical trials and observational studies of DOACs in AF with valvular heart disease demonstrated less risk of stroke and intracranial bleeding with DOACs, though these studies mostly included aortic valve disease, tricuspid valve disease and mitral regurgitation, with limited data for rheumatic mitral stenosis [20].

Although our real-world study showed no difference in MACCE or major bleeding between VKAs and DOACs in RHD-associated vAF, there were limitations. The small sample size limited the generalisability of our findings however it is important to note that the INVICTUS trial, with a much larger sample size, required a change in primary endpoint due to a low stroke event-rate in each arm. To have an adequately powered study (80 % with 95 % confidence interval and primary outcome incidence of ∼20 %), at least 79 patients per group would have been required to detect a statistically significant difference between groups. As a non-randomised cohort study, confounding and selection biases cannot be excluded. Patients were selected from a registry which may not capture all individuals with RHD in Central Australia. Our definition of vAF was in line with the INVICTUS trial and as a result, more inclusive of mild mitral stenosis than major clinical guidelines, creating a ‘low-risk’ cohort. Lack of adherence data is another important limitation. While we would expect adherence to be poorer for VKA therapy than DOAC in our population, adherence with VKA therapy actually exceeded DOAC in INVICTUS and so in the absence of data, we are unable to reliably predict the influence of adherence on the results.

Our retrospective pilot study demonstrated that DOACs may be a reasonable alternative to VKAs for reducing MACCE in patients with RHD-associated vAF in remote and resource-limited clinical contexts. There appears to be a trend towards use of DOACs over VKAs in this setting. Real-world challenges of prescribing VKAs mandate ongoing investigation of strategies to prevent MACCE and control RHD globally.

## CRediT authorship contribution statement

**Sonia Sawant:** Writing – review & editing, Writing – original draft, Methodology, Investigation, Data curation, Conceptualization. **Gabrielle Freedman:** Data curation. **Ana Garcia:** Data curation. **Sally Terrett:** Data curation. **Chinmay Khandkar:** Data curation. **Anosh Sivashanmugarajah:** Data curation. **Angus Baumann:** Writing – review & editing, Writing – original draft, Supervision, Resources, Methodology, Investigation, Formal analysis, Data curation, Conceptualization.

## Declaration of competing interest

The authors declare that they have no known competing financial interests or personal relationships that could have appeared to influence the work reported in this paper.
